# Transplacental transfer of glyburide in women with gestational diabetes and neonatal hypoglycemia risk

**DOI:** 10.1371/journal.pone.0232002

**Published:** 2020-05-07

**Authors:** Hanane Bouchghoul, Jean-Claude Alvarez, Céline Verstuyft, Jean Bouyer, Marie-Victoire Senat

**Affiliations:** 1 Department of Gynecology-Obstetrics, Assistance Publique-Hôpitaux de Paris, Bicêtre Hospital, Le Kremlin-Bicêtre, France; 2 Université Paris-Saclay, UVSQ, Inserm, CESP, Villejuif, France; 3 Département de Pharmacologie-Toxicologie, Assistance Publique-Hôpitaux de Paris, Hôpital Raymond Poincaré, MasSpecLab, Plateforme de spectrométrie de masse, Inserm U-1173, UFR PIFO, Université Versailles Saint Quentin-en-Yvelines, Garches, France; 4 Assistance Publique-Hôpitaux de Paris, Hôpital Bicêtre, Service de Génétique moléculaire, Pharmacogénétique et Hormonologie, Inserm U 1178 équipe Dépression, CESP, Université Paris-Sud, Le Kremlin-Bicêtre, France; Mount Sinai Health System, University of Toronto, CANADA

## Abstract

**Background:**

In pregnant women with gestational diabetes, glyburide can be an alternative to insulin despite concerns about its transplacental transfer. However, transplacental transfer of glyburide is poorly quantified and the relationship between cord blood glyburide concentration and hypoglycemia has not been studied. Our objective was to quantify the transplacental transfer of glyburide at delivery and to study the association between the cord blood glyburide concentration and the risk of neonatal hypoglycemia in patients with gestational diabetes treated with glyburide.

**Methods and findings:**

INDAO was a multicenter, noninferiority, randomized trial conducted between May 2012 and November 2016 in 914 women with singleton pregnancies and gestational diabetes. An ancillary study was conducted in the 87 patients of the Bicêtre University Hospital Center. The sample consisted of 46 patients with utilizable assays at delivery. The relationships between glyburide concentration and the time since the last intake of glyburide and between fetal glyburide concentration and neonatal hypoglycemia were modeled with linear or logistic regressions using fractional polynomials. There was placental transfer of glyburide at a fetal to maternal ratio of 62% (95% CI [50; 74]). Umbilical cord blood glyburide concentration decreased steeply after the last maternal glyburide intake. After 24 hours, the mean umbilical cord blood concentration was less than 5 ng/mL. Neonatal hypoglycemia risk was increased with an odds ratio of hypoglycemia equal to 3.70 [1.40–9.77] for each 10 ng/mL increase in the cord blood glyburide concentration. However, no newborns were admitted to the NICU because of clinical signs of hypoglycemia or for treatment of hypoglycemia.

**Conclusion:**

Considering that neonatal glyburide exposure may be limited by stopping treatment a sufficient time before labor, there may still be a place for glyburide in the management of gestational diabetes.

## Introduction

Gestational diabetes occurs in 9% to 25% of pregnancies, and its prevalence has increased over the last three decades due to the obesity epidemic [1–4]. The current recommended treatment in the case of failure of dietary intervention is insulin therapy. However, this treatment is costly and requires several injections per day, which can reduce compliance, so that oral anti-diabetic therapy such as glyburide has been proposed as an alternative.

Meta-analyses of randomized trials comparing glyburide to insulin have found increased macrosomia and neonatal hypoglycemia risks with glyburide [5–10], although these were not their primary criteria. Recently, the INDAO randomized noninferiority trial [11] suggested that the increase in neonatal complications is no more than 10.5% and concerns mainly neonatal hypoglycemia. Moreover, glyburide was associated with better glycemic control despite an increased risk of maternal hypoglycemia [11]. This leaves a potential role for glyburide therapy and calls for better understanding of its transplacental transfer.

An early study found no placental transfer of glyburide [12], but in this study the detection limit for glyburide was high and improvements in assay techniques in two more recent studies [13,14] showed that there is placental transfer with an estimated average ratio of umbilical cord to maternal blood glyburide concentration at delivery of 0.70 [13].

Neonatal hypoglycemia is usually considered to be a consequence of fetal hyperinsulinism induced by maternal hyperglycemia in the context of poorly controlled gestational diabetes [15]. However, it is unclear in pregnant women treated with glyburide whether neonatal hypoglycemia is associated with the placental transfer of glyburide.

The aims of this study were to describe and quantify, with current assay techniques, the transplacental transfer of glyburide at delivery in pregnant women treated with glyburide and to study the association with the risk of neonatal hypoglycemia.

## Material and methods

The study data were derived from an ancillary study of the INDAO (Insulin Daonil) randomized trial in which maternal and cord blood samples were collected to assess the transplacental transfer of glyburide [11].

INDAO was a multicenter, noninferiority, randomized trial conducted between May 2012 and November 2016 in 13 French tertiary care university hospitals in 914 women with singleton pregnancies and gestational diabetes diagnosed between 24 and 34 weeks of gestation [11]. Women who required pharmacologic treatment after 10 days of dietary intervention were randomly assigned to receive glyburide (n = 460) or insulin (n = 454).

The starting dosage for glyburide was 2.5 mg orally once per day and could be increased if necessary 4 days later by 2.5 mg and thereafter by 5 mg every 4 days in 2 morning and evening doses, up to a maximum of 20 mg/day. If the maximum tolerated dosage was reached without achieving the desired glucose values of less than 95 mg/dL for fasting measurements and less than 120 mg/dL for 2-hour postprandial measurements, treatment was switched to insulin. Women were advised not to take their glyburide treatment when labor was approaching. The treatment was stopped upon admission to the delivery room hourly monitoring of capillary blood glucose was implemented.

Management consisted of self-monitoring 4 times a day, with blood glucose targets of less than 95 mg/dL for fasting and less than 120 mg/dL for 2-hour postprandial values. Each woman had a blood glucose meter and a notebook to record blood glucose levels, which she filled out prospectively. The notebook was made available for evaluation of glycemic control (separately for fasting and postprandial blood glucose) by the percentage of blood glucose levels outside the target range. Good glycemic control was defined as a percentage below 20%.

An ancillary study was conducted in the Bicêtre University Hospital Center. All the patients randomized to the glyburide group in this center and still receiving glyburide at delivery were invited to participate. The study consisted of sampling 5 mL of blood from the mother and 5 mL of cord blood at the time of delivery. The blood sample assays were performed by liquid chromatography coupled to tandem mass spectrometry. Intra- and inter-assay coefficient of variation was 1 ng/mL, and the method was linear between 1 and 500 ng/mL. Intra- and inter-assay coefficients of variation evaluated at 3, 30 and 300 ng/mL were less than 6.5% [16]. In addition to maternal and umbilical cord blood glyburide concentrations, the variables recorded included: general characteristics of the woman (age, parity, weight gain, history of diabetes), daily glyburide dosage received by the woman, time between delivery and the last intake, neonatal outcomes such as macrosomia (defined as birth weight greater than 4000 g or above the 90th percentile for gestational age), hypoglycemia (defined as capillary blood glucose less than 36 mg/dL), hyperbilirubinemia (defined as the need for phototherapy without another cause of jaundice).

Monitoring of newborns was identical to what is usually recommended for newborns of mothers with diabetes. Capillary blood glucose was measured before the first feeding, before the second feeding, and then every 3 hours, before feeding, for asymptomatic newborns. Hypoglycemia was defined in the analysis as blood glucose below 36 mg/dL (<2 mmol/L) after 2 hours of life.

Transplacental transfer of glyburide was quantified by the ratio of umbilical cord over maternal blood concentration [17].

The INDAO trial protocol, including this ancillary study for which patients provided additional informed consent, was approved by the ethics committee of the Poissy St-Germain Hospital (France) and registered at clinicaltrials.gov (Identifier NCT01731431). Data were collected by research staff members from medical records including medical history and outcome data.

For the statistical analysis, we first studied the relationship between maternal and fetal glyburide concentrations and quantified the transplacental transfer of glyburide. Then, we modeled the relationship between maternal glyburide concentration and the time since the last intake of glyburide. Finally, we modeled the association between fetal glyburide concentration and neonatal hypoglycemia. Quantitative variables were described with mean and standard deviation (SD) or median and IQR (interquartile range) according to their distribution. The relationships between outcomes (cord or maternal blood glyburide concentration and neonatal hypoglycemia) and independent variables (which may be depending on the case the time period since the last oral glyburide intake or cord or maternal blood glyburide concentration) were modeled with linear or logistic regressions using fractional polynomials, which give an optimal data fit [18]. The statistical analyses were performed with Stata 14 [19].

## Results

**Fig 1:**
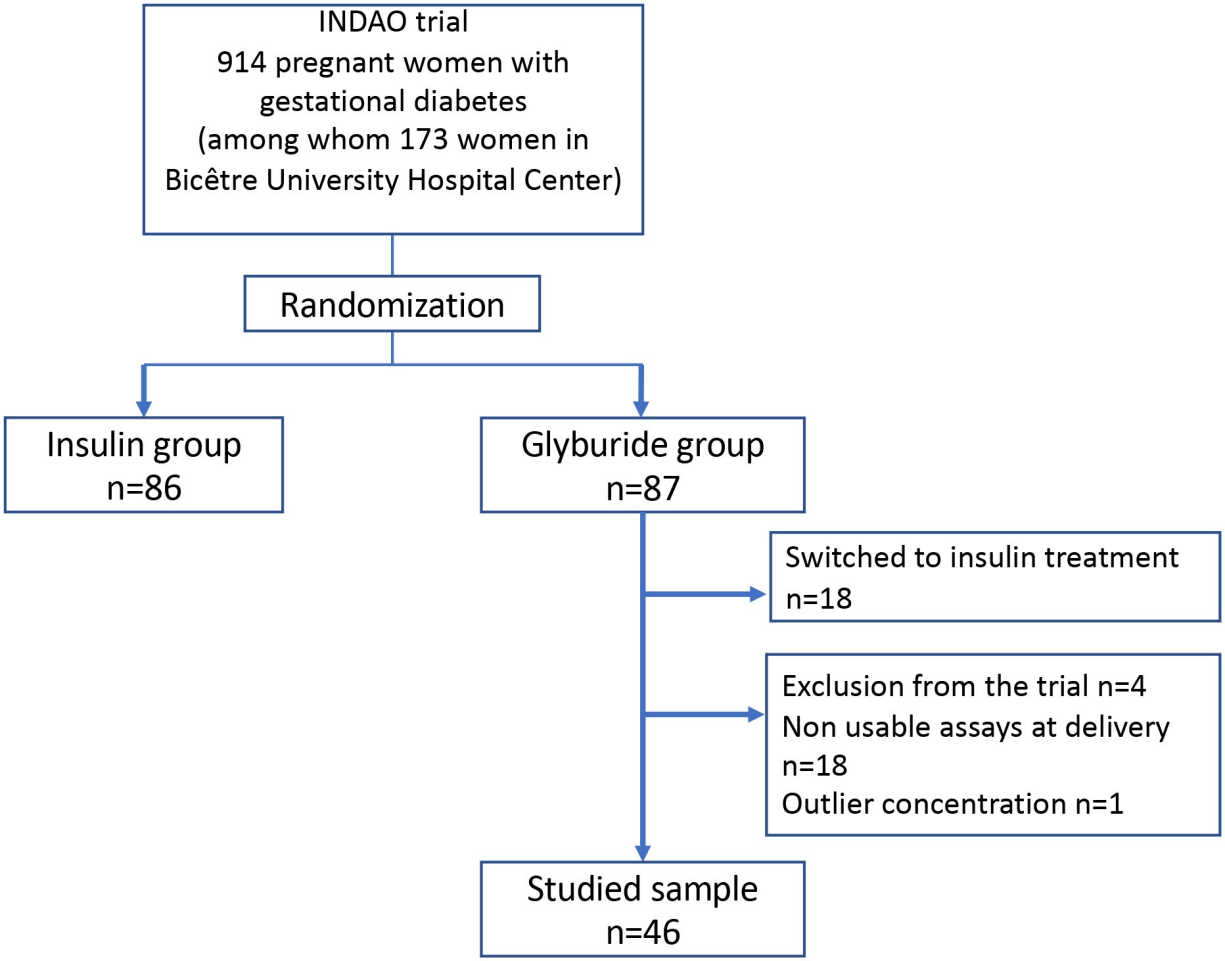
Flow chart.

Of 914 patients included in the INDAO randomized trial, 173 were in the Bicêtre University Hospital Center. Of these 173 patients, 87 were randomized to the glyburide group (Fig 1): 4 patients were excluded from the trial (type 2 diabetes, pharmacotherapy unnecessary, lost to follow-up, treatment refused), and 18 patients were switched to insulin treatment due to inadequate blood glucose control. Finally, 65 patients were eligible for the study, of whom 18 had no available sample. Moreover, 1 patient was excluded because of isolated outlier concentrations despite the checking of the assays (100 ng/mL in umbilical cord blood and 150 ng/mL in maternal blood); the newborn of this patient did not present hypoglycemia. Finally, the sample consisted of 46 patients with utilizable assays at delivery.

The general characteristics of the 46 remaining patients are given in Table 1. The 19 patients who were eligible but either had no blood sample or an unusable blood sample (and were thus excluded) had similar characteristics. In particular, the quality of the glycemic control during pregnancy was similar in these two groups.

**Table 1:** General characteristics of the 46 patients treated with glyburide.

Characteristics	Sampling, n = 46
Age (years)	30.9 (5.0)
Multiparity	31 (67.4%)
Pre-pregnancy BMI (kg/m^2^)	26.1 (3.7)
Weight gain during pregnancy (kg)	11.6 (5.6)
Previous gestational diabetes	9 (19.6%)
Gestational age at diagnosis, (weeks^+days^)	27^+0^ [25^+3^–27^+6^]
Gestational age at randomization, (weeks^+days^)	32^+1^[31^+0^–34^+1^]
Glycemic control during pregnancy	
Good fasting blood glucose *	35/42 (83.3%)
Good postprandial blood glucose ^†^	24/42 (57.1%)
Gestational age at delivery (weeks^+days^)	38^+0^[37^+5^–39^+0^]
Mode of delivery	
Spontaneous labor	14 (30,4%)
Induction of labor	29 (63,0%)
Planned Cesarean section	3 (6,6%)

Data are n (%), mean (sd), or median [interquartile range] unless otherwise specified.

BMI: body mass index

* good fasting blood glucose is defined as less than 20% of fasting blood glucose measurements > 95 mg/dL

^†^ good postprandial blood glucose is defined as less than 20% of fasting blood glucose measurements > 120 mg/dL

Table 2 shows the glyburide concentrations in umbilical cord plasma and in maternal plasma at delivery. The mean ratio between cord and maternal blood concentration was 0.62 (95% CI [0.50–0.74]). Last glyburide dose intake before delivery was significantly and positively associated with cord blood glyburide concentration (result not shown, p<0.01).

**Table 2:** Pharmacologic characteristics of cord and maternal blood samples, n = 46.

Characteristic	Median [IQR*]
Maternal blood glyburide concentration at delivery (ng/mL)	9.9 [3.7–27.0]
Cord blood glyburide concentration at delivery (ng/mL)	6.8 [2.1–14.8]
Ratio between cord blood and maternal plasma glyburide concentrations	0.62 (0.50–0.74)**
Daily dosage of glyburide at the end of pregnancy (mg)	7.5 [3.25–10.0]
Time since the last oral glyburide intake (hours)	16.0 [10.5–24.2]

* Inter Quartile Interval

** mean (95% CI)

**Fig 2:**
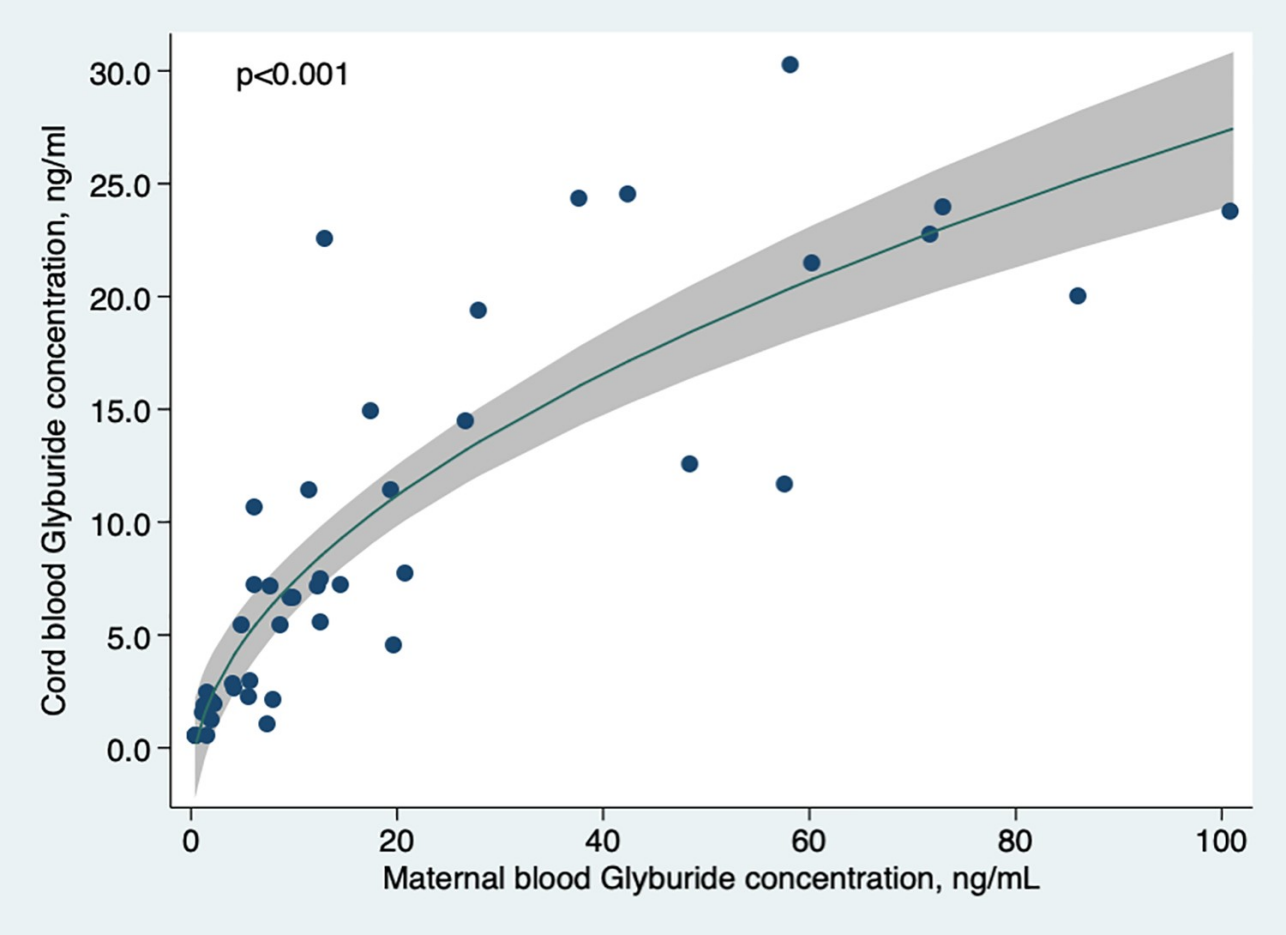
Cord blood glyburide concentration according to maternal blood concentration at delivery. Curve was modeled using fractional polynomials.

There was a strong and significant (p<0.001) relationship between umbilical cord and maternal blood concentration (Fig 2).

**Fig 3:**
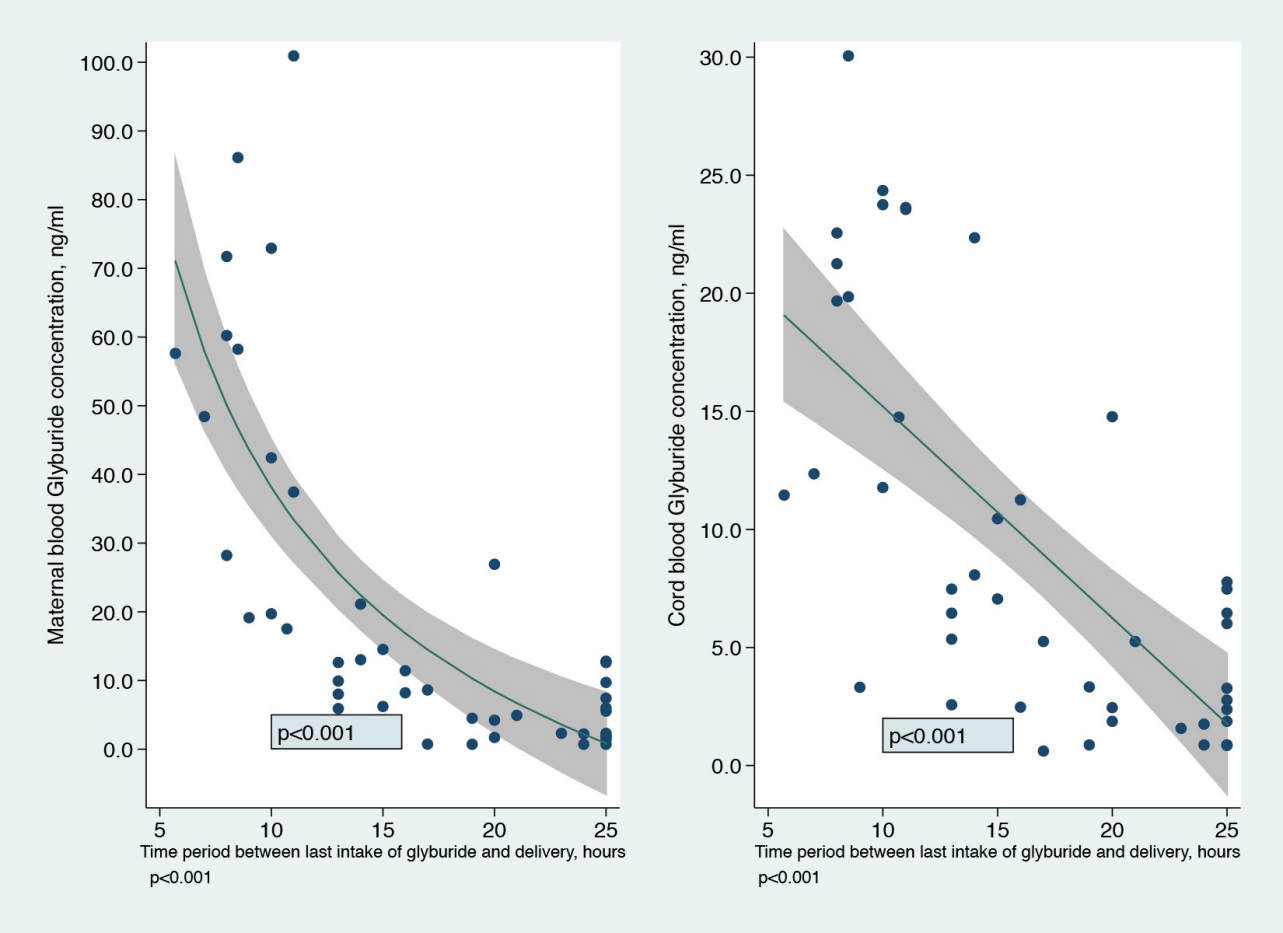
Cord blood and maternal blood glyburide concentrations at delivery according to the time period since the last oral glyburide intake. Curves were modeled using fractional polynomials and adjusted for the last dose of glyburide. They were drawn for the average value of the last dose of glyburide.

The median time elapsed since the last oral glyburide intake at delivery was 16.0 hours (IQR [10.5–24.2]). After adjustment for the last glyburide dose intake, there was a large and significant (p<0.001) decrease in umbilical and maternal blood concentration with time since the last dose (Fig 3). After 24 hours, the mean umbilical cord blood concentration was less than 5 ng/mL.

**Fig 4:**
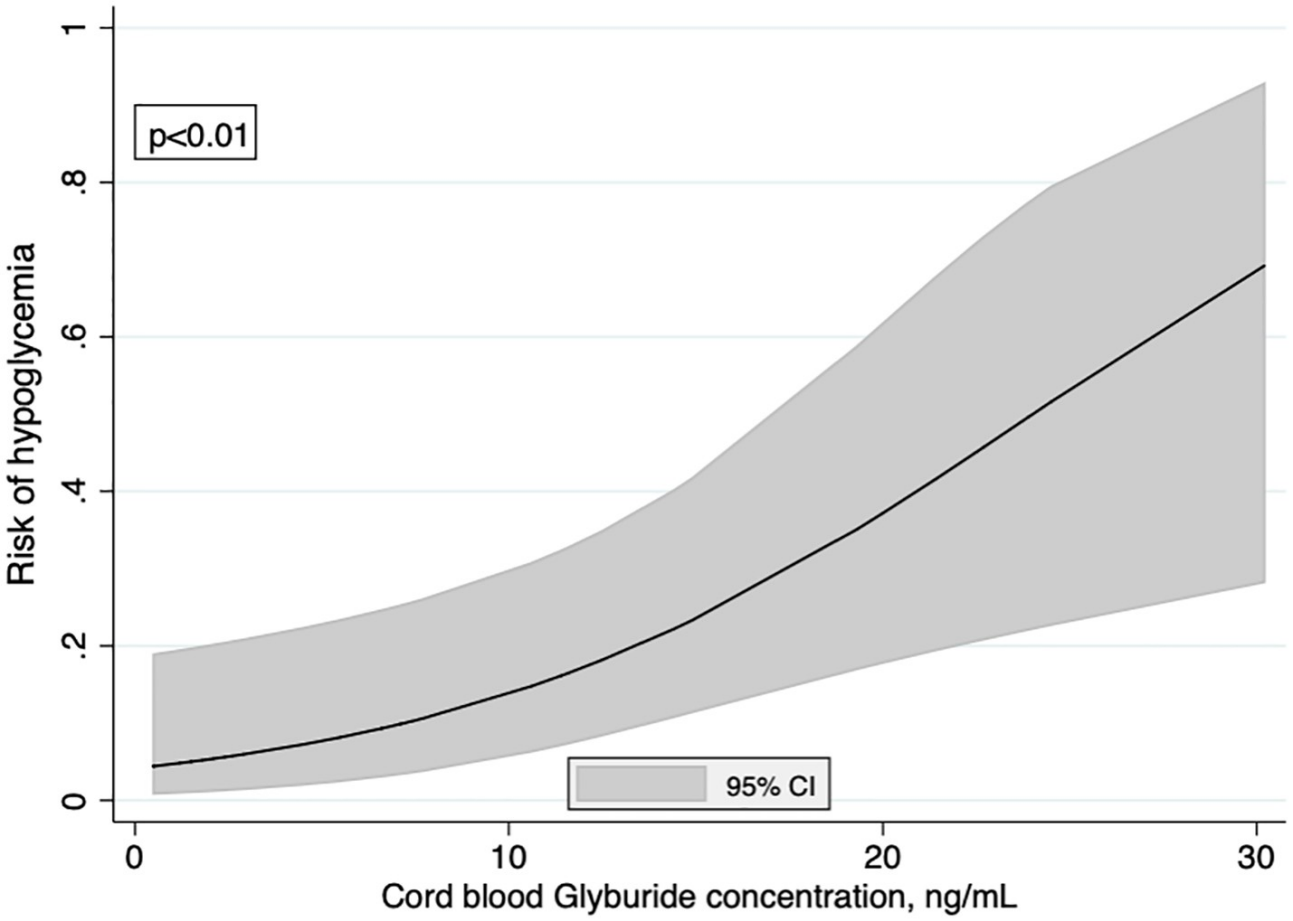
Risk of hypoglycemia according to the cord blood glyburide concentration. Curve was modeled using fractional polynomials.

The proportion of neonatal hypoglycemia was 17.4% (95% CI [0.08–0.31]) in the 46 neonates of the sample and increased significantly according to the cord blood glyburide concentration (p<0.01) (Fig 4). The fractional polynomial showed a linear relationship (in logit unit) that could be summarized by an odds ratio (OR) of hypoglycemia equal to 3.70 [1.40–9.77] for each 10 ng/mL increase in the cord blood glyburide concentration. This strong relationship was unchanged after adjustment for macrosomia (adjusted OR = 4.65 [1.36–15.94], p = 0.01). However, no neonate developed severe clinical signs of hypoglycemia. No newborns were admitted to the NICU because of hypoglycemia or for treatment of hypoglycemia.

## Discussion

Our findings confirm the placental transfer of glyburide, with a fetal to maternal ratio of 62% (95% CI [50–74]). The umbilical cord blood glyburide concentration decreased steeply after the last maternal glyburide intake. The risk of neonatal hypoglycemia increased significantly with umbilical cord blood glyburide concentration, independently of fetal macrosomia at birth. Moreover, no neonate presented severe clinical signs of hypoglycemia.

Our study is the first to look at the entire process from glyburide intake to cord blood concentration and then to assess the risk of neonatal hypoglycemia with assay techniques much more sensitive than those used in studies [12]. However, despite the short elimination half-life of glyburide (about 4 to 10 hours in pregnant women [20,21]), pharmacokinetic data could not be evaluated since this required regular blood sampling over time. Potential limitations of this study are the sample size and the non-availability of 19 assays.

Glyburide is a second-generation hypoglycemic sulfonamide used to treat type 2 diabetes by stimulating insulin secretion by β-pancreatic cells. The pharmacokinetic properties of glyburide are modified in a patient with gestational diabetes during pregnancy [13]. Hebert et al compared the pharmacokinetic properties of glyburide between 40 women treated for gestational diabetes during pregnancy and 40 women treated with type 2 diabetes outside of pregnancy. They found that the maternal blood concentration was half as low during pregnancy. After oral administration, clearance of glyburide was greater in pregnant women (17.1 L/hour) than in non-pregnant women (8.3 L/hour).

Our results are consistent with two previous studies of the transplacental transfer of glyburide [13,14]. In 2009, Hebert et al published the first study to evaluate transplacental transfer and reported a ratio of 0.70 for fetal to maternal blood glyburide concentration at birth in 40 patients with gestational diabetes treated with glyburide [13]. Schwartz et al found a weak inverse relationship between cord blood glyburide concentration and neonatal blood glucose levels at 30–40 minutes of life [14].

Our study confirms that glyburide does cross the placental barrier. Because of a greater sample size than previous studies, our study also provides an accurate estimate of the ratio between umbilical cord and maternal blood concentrations. The shape and strength of the relationships were evaluated more accurately since they were modeled with fractional polynomials. In particular, we have highlighted the importance of the time elapsed since the last maternal intake of glyburide. After 24 hours, the umbilical cord blood concentration was less than 5 ng/mL. Combined with the relationship between umbilical cord blood glyburide concentration and risk of hypoglycemia, this suggests that the risk of neonatal hypoglycemia may be reduced to less than 15% if the time since the last intake is extended to 24 hours.

Other assays, of C-peptide for example, could also shed light on the mechanism of hypoglycemia. An elevated C-peptide concentration would be consistent with stimulation of pancreatic β-cells and so with fetal stimulation by glyburide.

Our study opens up the possibility that neonatal hypoglycemia could be reduced by stopping glyburide as soon as possible, and ideally 24 hours before onset of labor. This can be achieved by advising the woman not to take her treatment or by switching to insulin when she is hospitalized for an upcoming delivery. However, the concern remains that fetal exposure to glyburide may have an effect on the potential long-term morbidity associated with the child's endocrine axis.

Many factors, which deserve further study, are involved in maternal blood glyburide concentration and therefore in fetal exposure to glyburide. There are inter-individual variations which can be explained by genetic polymorphisms for CYPs in women because glyburide is partially metabolized by the liver, with the involvement of cytochrome P450 2C9 [22]. Carriers of the CYP2C9 variant have decreased oral clearance of glyburide. Thus, the genetic polymorphism of CYP2C9 could influence glyburide concentrations and side effects like neonatal hypoglycemia. Moreover, Kraemer et al published the first study to show the existence of placental efflux against a gradient concentration using a placental perfusion model [23]. Placental transporters (ABC transporters = ATP binding cassette) have an important role in fetal drug exposure and may therefore account for this efflux process [24,25]. However, the inter-individual variability of placental expression of these proteins has not been studied.

Finally, some authors have shown that human fetal liver metabolizes glyburide and that CYP3A7 is the major enzyme responsible for this metabolism [26]. They also showed that microsomal CYP3A7 protein content in human fetal liver was not affected by fetal sex, genotype, or gestational age. Further work is needed on the long-term outcome of children of diabetic mothers treated with glyburide during pregnancy.

### Conclusions

Due to placental transfer of glyburide, its administration in the few hours before delivery was associated with a risk of neonatal hypoglycemia, which, however, was mild and reversible. This risk may be limited in some cases by stopping treatment in sufficient time delay before delivery (planned cesarean section, induction of labor).
